# Use of Cusp Catastrophe for Risk Analysis of Navigational Environment: A Case Study of Three Gorges Reservoir Area

**DOI:** 10.1371/journal.pone.0158482

**Published:** 2016-07-08

**Authors:** Dan Jiang, Guozhu Hao, Liwen Huang, Dan Zhang

**Affiliations:** 1 Department of Navigation, Wuhan University of Technology, Wuhan, Hubei, China; 2 Key Laboratory of Hubei Inland Shipping Technology, Wuhan, Hubei, China; 3 Department of Navigation, Chongqing Jiaotong University, Chongqing, China; Shanxi University, CHINA

## Abstract

A water traffic system is a huge, nonlinear, complex system, and its stability is affected by various factors. Water traffic accidents can be considered to be a kind of mutation of a water traffic system caused by the coupling of multiple navigational environment factors. In this study, the catastrophe theory, principal component analysis (PCA), and multivariate statistics are integrated to establish a situation recognition model for a navigational environment with the aim of performing a quantitative analysis of the situation of this environment via the extraction and classification of its key influencing factors; in this model, the natural environment and traffic environment are considered to be two control variables. The Three Gorges Reservoir area of the Yangtze River is considered as an example, and six critical factors, i.e., the visibility, wind, current velocity, route intersection, channel dimension, and traffic flow, are classified into two principal components: the natural environment and traffic environment. These two components are assumed to have the greatest influence on the navigation risk. Then, the cusp catastrophe model is employed to identify the safety situation of the regional navigational environment in the Three Gorges Reservoir area. The simulation results indicate that the situation of the navigational environment of this area is gradually worsening from downstream to upstream.

## Introduction

Since the impoundment of the Three Gorges Reservoir, the navigation condition of the reservoir area has greatly improved, which has promoted the development of shipping in this area. However, the expansion of the navigable region has resulted in an increasing number of new problems in the reservoir area. First, the increase in the water surface has exacerbated moisture evaporation. This has led to heavy fog, which adversely affects the lookout effectiveness on ships. Second, the rise in the water level has caused the submergence of nautical marks, which hampers the provision of the guidance needed for ship sailing. Third, an explosion in the number of vessels has increased the ship density within the reservoir area, which has increased the complexity of ship collision avoidance planning. Thus, these new modifications in the navigational environment in the Three Gorges Reservoir area have caused it to become a high-risk area for ship navigation.

From the viewpoint of system theory, a water traffic system is a huge, nonlinear, complex system that entails interactions among humans, ships, and the navigational environment. The evolvement rules of a water traffic system are characterized by a highly nonlinear dynamics mechanism. The navigational environment, as an important component of the water traffic system, affects not only water traffic safety but also the factors pertaining to humans and ships, which may also result in an increased probability of accidents [1–5]. A stable navigational environment can ensure navigation safety to a considerable extent, whereas a mutation of the navigational environment from stable to unstable may cause significant problems in ship navigation, or even maritime accidents. Thus, research on the influence of the navigational environment on water traffic safety is of great significance.

The overall impact of a variety of environmental factors on ship navigation safety is an indication of the safety situation of the navigational environment. At present, the safety situation of a navigational environment is judged mainly by an evaluation of ship navigation safety and accident analysis [6–10]. Specialized studies focused on the navigational environment have rarely been conducted. Most of these studies have treated the navigational environment as a single entity in a qualitative analysis; quantitative calculations of and comparative research on the navigational environment have rarely been conducted.

In previous studies, evaluations of the degree of safety of some regional navigational environments were based mostly on the establishment of an evaluation index system. From the perspective of the physical space, these evaluation systems were all 2D models of related parameters.

Nonlinear system theories such as dissipative structure theory, catastrophe theory, and nonlinear dynamics theory are powerful tools to study a nonlinear navigation system. Catastrophe theory is used in the study of the external control condition when mutation occurs to a system state and mainly describes how a nonlinear system tends toward catastrophe from a state of continuous gradual change. Given this background, on the basis of the particularity of the environmental changes in the Three Gorges Reservoir area, we here use multivariate statistical methods and mutation theory to construct a safety identification model for the navigational environment of this area and provide a 3D illustration of the normal or abnormal combination relationships between various environmental factors in different segments of the Three Gorges Reservoir area. The establishment of an overall picture of the situation of the navigational environment of the reservoir area through the acquisition of the factors of this environment can enable the provision of guidance for maritime supervision and further improve the early warning provision and emergency management capabilities for this navigational area, so as to reduce accidents and promote navigation safety.

## Fundamentals of Catastrophe Theory

Catastrophe theory is a branch of nonlinear theory that uses a mathematical model to discuss the common law for rapid changes of state in a dynamic system. It mainly explains how continuous changes in parameters lead to a discontinuous phenomenon. Catastrophe theory was derived from mathematics and developed on the basis of topology and the theories for the singularity and stability of a system structure. Catastrophe theory mainly studies the sudden changes in a dynamic system in the process of sequential development and explains the relationship between these sudden changes and succession factors. It is not constrained by exposing of the internal complex feedback and function mechanisms, but is used to control the phenomenon of catastrophe by an analysis of multiple governing factors that affect the occurrence of mutations [11–14]. From the viewpoint of mathematical analysis, catastrophe theory is implemented through a study of the potential function of a system, which can be described as in Eq 1:

V=V(x,c) (1)

where *x* and *c* are the state variable and control variable, respectively, of a system. When a system has no more than four control variables, the number of elementary catastrophe models is seven. Among these, basic catastrophe models with one state variable and four control variables are presented in Table 1.

**Table 1:** Primary Mutation Models and Corresponding Potential Functions.

Mutation model	Number of control variables	Number of state variables	Dimensions of phase space	Standard potential function
**Fold catastrophe**	1(*u*)	1(*x*)	2	*V*(*x*) = *x*^3^ + *ux*
**Cusp catastrophe**	2(*u*,*v*)	1(*x*)	3	*V*(*x*) = *x*^4^ + *ux*^2^ + *vx*
**Swallowtail catastrophe**	3(*u*,*v*,*w*)	1(*x*)	4	*V*(*x*) = *x*^5^ + *ux*^3^ + *vx*^2^ + *wx*
**Butterfly catastrophe**	4(*u*,*v*,*w*,*t*)	1(*x*)	5	*V*(*x*) = *x*^6^ + *tx*^4^ + *ux*^3^ + *vx*^2^ + *wx*

## Cusp Catastrophe Model of Navigational Environment

### Selection of control variables and classification

The mutation of the navigation risk is a result of the comprehensive interaction of multiple factors [15,16]. Therefore, in the process of using a mutation model for simulation, the first task is to determine the subfactors that cause navigation risk mutations.

Given the task of reflecting the synthesized state of the navigational environment of the Three Gorges Reservoir area from different perspectives and making the selection of an appropriate index for the goal of navigation safety, we found that six key navigational environment factors affect the safety of navigation in the Three Gorges Reservoir area, i.e., the visibility, wind power, traffic flow, channel dimension, and route intersection. These factors were determined through an analysis of the cause of maritime accidents in the Three Gorges Reservoir area, in combination with a survey of staff in the maritime industry, pilots, and experts in maritime colleges. Thus, we selected these six factors as indicators in the navigational environment of the Three Gorges Reservoir area.

According to the mutation model of the navigational environment, the variables of visibility (*x*_*1*_), wind (*x*_*2*_), and water velocity (*x*_*3*_) are classified as control variables of the natural environment (*M*), whereas the variables of traffic flow (*x*_*4*_), channel dimension (*x*_*5*_), and route intersection (*x*_*6*_) are classified as control variables of the traffic environment (*N*). Thus, the navigation risk mutations caused by the navigational environment can be summarized as coupling mutations generated from these two sets of control variables. In the cusp catastrophe model, the two sets of control variables have a logical progression. The primary control variable is the ripping factor, which is the coefficient of *x*^*2*^, and the secondary control variable is a regular factor, which is the coefficient of *x*. The application of an expert scoring method and fuzzy statistics reveals that the natural environment makes a greater contribution to the navigation risk than the traffic environment [17].

### Establishment of cusp catastrophe model

The cusp catastrophe model involves one state variable *X* and two control variables *M* and *N*. Let *X* be the security-state variable of the navigational environment system, and let the potential function *V* reflect the ability of the water traffic safety system to maintain stable operation. The standard form of the potential function *V* can be expressed as follows:

$$V(X)=X^{4}+MX^{2}+NX$$ (2)

where *X* is the state variable of the navigational environment system, and *M* and *N* are the two control variables of the system.

The balance curved surface is given by the following:

$$4X^{3}+2MX+N=0$$ (3)

The curved surface is called the catastrophe manifold; it represents the conditions met by the balanced state of the system, and its corresponding point is called a critical point in mathematics [13]. All of the points on this surface (*X*, *M*, *N*) are referred to as the balance points of the potential function *V*. The equation of singular points can be written as follows:

$$12X^{2}+2M=0$$ (4)

If we project singular points onto the control surface *M-N*, the branch points *B* can be expressed as follows:

$$8M^{3}+27N^{2}=0$$ (5)

**Fig 1:**
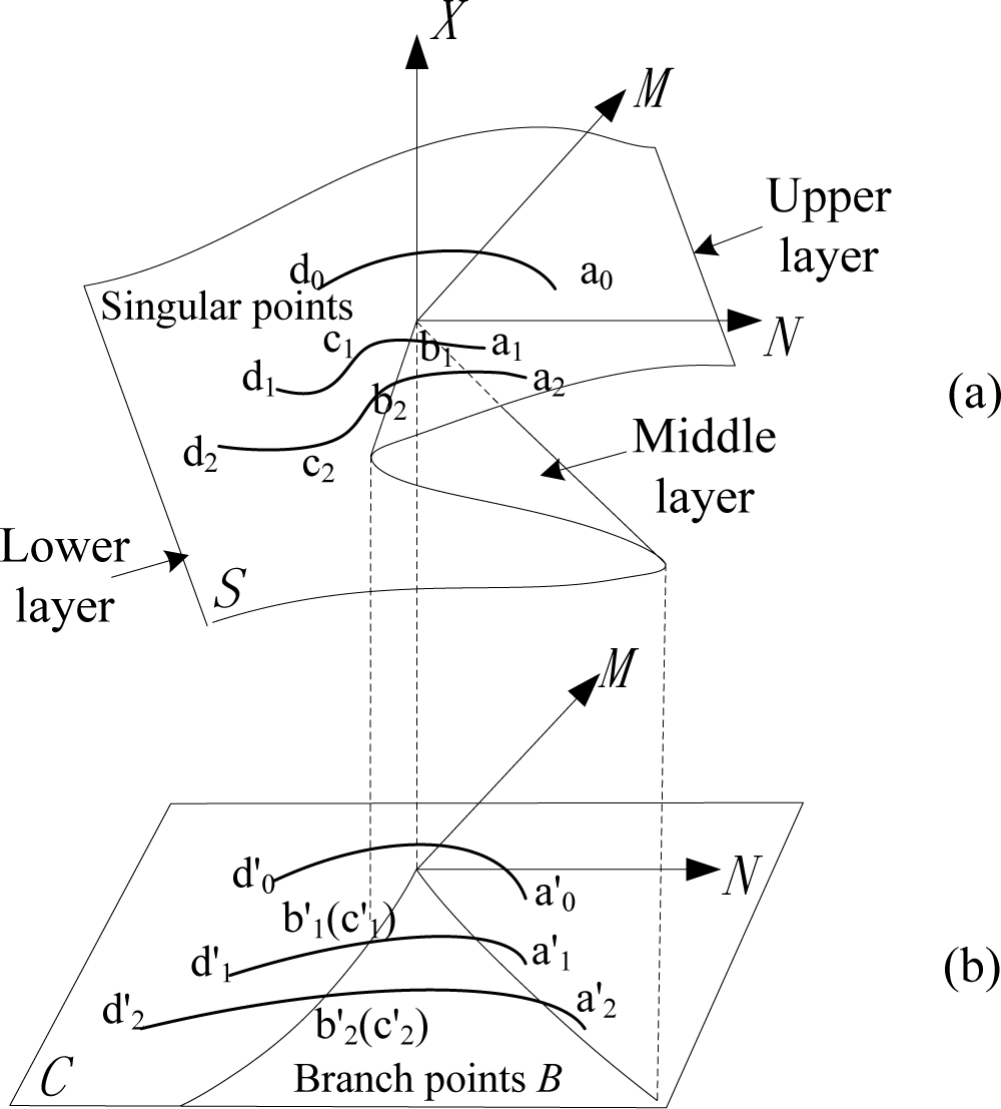
Evolution Model of Navigation Risk with Variation of Navigational Environment. *S* is the balance surface and *C* is the *M-N* control surface.

Changes in the control variables *M* and *N* essentially cause stable changes in the state variable *X*. A discontinuous catastrophe will occur only when the controlling points (*M*, *N*) jump over a branch point *B*. The points located on the two branches of *B* are singular points of the system, i.e., catastrophe points [18], as shown in Fig 1.

Under the influence of the natural and navigational environments, the navigation safety system usually evolves from a balanced state to a state of imbalance. When the system is in a critical condition, it is extremely sensitive to external disturbances. A small disturbance would probably induce a catastrophe in the navigation system.

### Analysis of mutation mechanism

The evolution pattern of the navigation risk with the variation of the navigational environment is shown in Fig 1.

In Fig 1, the upper layer reflects the harmonious orderly situation of safe navigation, the lower layer reflects the accident state, and the middle layer represents the unstable mutation region that is inaccessible. The three different curves indicate the evolution of the mutation of navigation risk and the cause of a water traffic accident under the combined action of the natural environment factors and traffic environment factors.

- The curve $a_{0}^{\prime }d_{0}^{\prime }$ does not pass the cusp manifold area, which refers to a smooth transition from the upper layer to the lower layer of the surface of the potential function. The navigation situation changes gradually and there is a serious probability of an accident in the system. However, even if the navigational environment worsens, it will not lead to a water traffic accident.
- Both the curves $a_{1}^{\prime }b_{1}^{\prime }c_{1}^{\prime }d_{1}^{\prime }$ and $a_{2}^{\prime }b_{2}^{\prime }c_{2}^{\prime }d_{2}^{\prime }$ pass through the fold area of the surface of the potential function. The phase potential of the system transforms suddenly from *b*_1_ → c_1_ and *b*_2_ → c_2_. This process indicates that changes in the control variables will result in the occurrence of a water traffic accident.
- The variations of the phase potential of the navigational environment system during the period of the two controlling curves passing through the fold area are Δ*X*_1_ = *X*(*M*_*b*1_,*N*_*b*1_)−*X*(*M*_*c*1_,*N*_*c*1_) and Δ*X*_2_ = *X*(*M*_*b*2_,*N*_*b*2_)−*X*(*M*_*c*2_,*N*_*c*2_), respectively. Evidently, Δ*X*_1_<Δ*X*_2_, which represents the consequence and damage extent of a water traffic accident as derived from the curve *a*_2_*b*_2_*c*_2_*d*_2_, is more serious than the curve *a*_2_*b*_2_*c*_2_*d*_2_.

### Solution of mutation area of cusp catastrophe model

**Fig 2:**
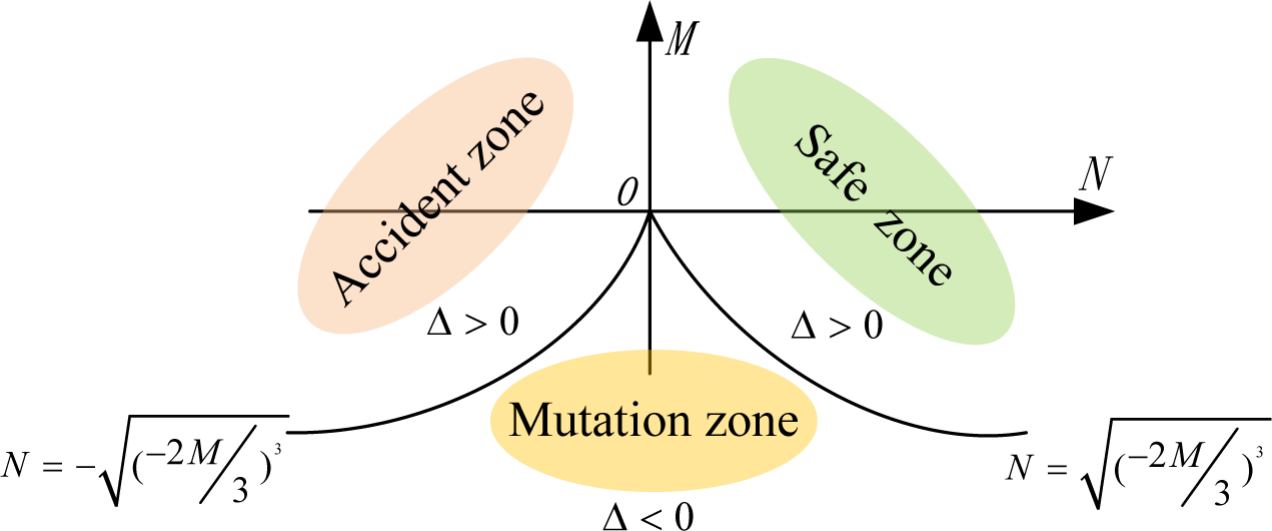
Cusp Mutation Area of Navigational Environment.

Determination of the cusp area is the key to judging the situation of the navigational environment. Here, Δ = 8*M*^3^ + 27*N*^2^ is the criterion for deciding whether or not the situations corresponding to various control intervals are stable. In order to confirm the boundary of the mutation area, we simply need to make Δ = 0, i.e., 8*M*^3^ = -27*N*^2^; in other words, any *M* and *N* that satisfy the equation N=±(−2M3)3 lie on the edge of the mutation area of the cusp catastrophe model [19,20]. The distribution relationship between the situations of the navigational environment and the control variables is shown in Fig 2.

When Δ < 0, Eq 3 has three different roots, and the points (*M*, *N*) satisfying the condition are in the cusp area; that is, the navigation system is in an unstable state and may have mutations.

When Δ > 0, Eq 3 has only one root, and all the points (*M*, *N*) satisfying the condition are outside the cusp area, indicating that the system is stable.

When Δ = 0, Eq 3 has two roots, and the points (*M*, *N*) lie on the edge of the mutation area; that is, the system is in a critical condition. In particular, when *M* = *N* = 0, both the roots are equal to zero, and the point (*M*, *N*) lies right on the cusp.

## Evaluation of Navigational Environment Risk Based on Catastrophe Theory

**Fig 3:**
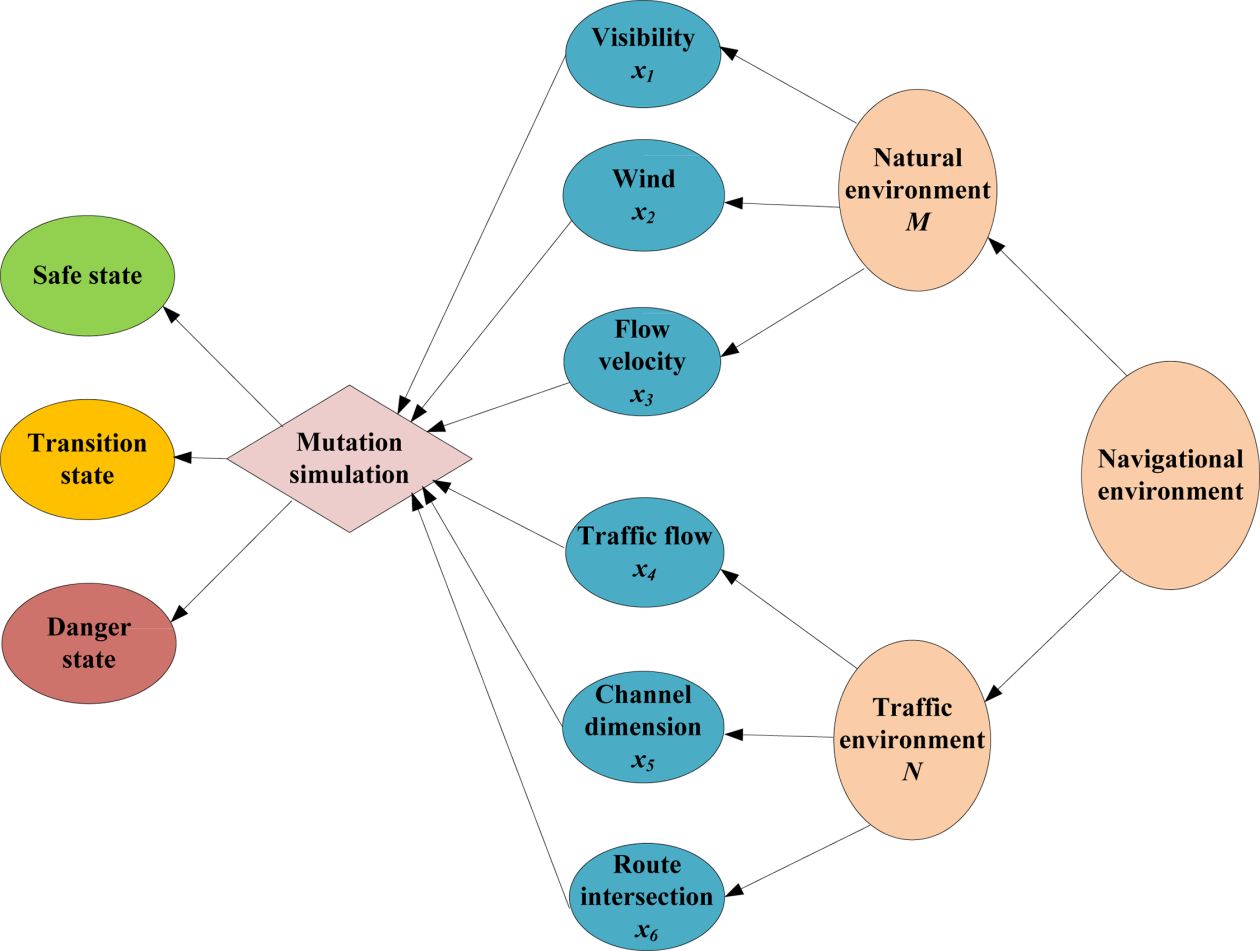
Framework for Evaluation of Navigation Risk.

Since the impoundment of the Three Gorges Reservoir area, the channel conditions have improved greatly. However, new problems pertaining to the navigational environment, such as frequent heavy fog and an increase in vessel density, are occurring increasingly frequently. The navigational environment can be classified into two kinds. One is the traffic environment, which is composed of beacons, traffic flow, anchorage, etc. The other is the natural environment, with wind, current, visibility, and water level as the key factors. The mutations of the navigation risk and water traffic accidents arising from the variation of the navigational environment can be regarded as being caused by the combined action of the traffic environment and natural environment. The framework for the evaluation of the navigation risk is shown in Fig 3.

### Data acquisition for navigational environment in Three Gorges Reservoir area

#### Selection of sample collection sites

For the purpose of a comprehensive evaluation of the navigational environment situation in various water areas, we collected data from each sector of the Three Gorges Reservoir area. We selected 13 hydrology, meteorology, and traffic monitoring stations along the Yangtze River in the Three Gorges Reservoir area as data collection sites. Because we collected only essential data for analysis purposes and did not damage the sites in any way, no specific permissions were required for accessing these sites.

The locations of the sites from the head of the reservoir to the west are, in order, Yichang, Guizhou, Badong, Wushan, Fengjie, Yunyang, Wanzhou, Zhongxian, Fengdu, Fuling, Changshou, Chongqing, and Jiangjin. Based on the channel mileage statistics of each site, we could establish that the distances between Fengjie and Yunyang, between Wanzhou and Zhongxian, and between Changshou and Chongqing are large: about 80 km. The channel distances of the remaining sites are approximately 40 km. The distribution of the selected sites along the Yangtze River was essentially uniform, and the collected data can completely illustrate the status of the navigational environment in the Three Gorges Reservoir. The location parameters of each sample site are presented in Table 2.

**Table 2:** Channel Mileage Statistics of Data Collection Sites in Three Gorges Reservoir Area.

Data collection site		Upstream channel mileage
**From downstream to** u**pstream**	**Yichang**	50
	**Guizhou**	80
	**Badong**	123
	**Wushan**	168
	**Fengjie**	216
	**Yunyang**	290
	**Wanzhou**	337
	**Zhongxian**	419
	**Fengdu**	487
	**Fuling**	548
	**Changshou**	585
	**Chongqing**	657
	**Jiangjin**	725

The Yangtze River is divided into three parts in order: upstream, middle stream, and downstream. The upstream channel mileage refers to the distance from Zhongshui Gate, and the unit is kilometers.

#### Collection of original metrical data

The original variables obtained using objectively measured data can reflect the true state of a system. The values of variables such as the wind, current, and traffic flow could be acquired in this way. The wind factor was measured using the annual mean of typical wind days. The current was measured using the maximum velocity of the water flow in the channel. Finally, the factor of traffic flow was measured using the daily average number of vessels for the years 2011–2013. These data were provided by the Yangtze River Maritime Safety Administration, Three Gorges Authority, Chongqing Maritime Bureau, and Chongqing Hydrographic Department. Nearly two months were required to visit these institutions and gain access to these original data (see Table 3).

**Table 3:** Collection of Original Metrical Data.

Sample collection site	Wind (days)	Flow velocity (m/s)	Traffic flow (ships per day)
**Yichang**	47	1.4	470.5
**Guizhou**	43	2.4	395.2
**Badong**	44.5	3.0	322.5
**Wushan**	38	3.4	237.5
**Fengjie**	45.5	2.8	230.6
**Yunyang**	35	3.3	228.7
**Wanzhou**	33	3.5	248.8
**Zhongxian**	45	3.6	236.9
**Fengdu**	36	3.9	237.5
**Fuling**	30	4.3	246.1
**Changshou**	34.5	4.5	232.6
**Chongqing**	26	5.0	249.3
**Jiangjin**	25	5.0	179.6

#### Collection of quantized data

To estimate the impact of visibility on the navigation safety, factors such as the intensity of rainfall and the intensity and duration of fog should be considered systematically. The channel dimension is affected by natural factors such as the water level variation and sediment deposition, and it is reflected in the channel width, water depth, bending radius, height clearance, and obstruction. In addition to the number of intersecting routes and the crossing angles of the routes, the route intersection is also related to the traffic flow of each channel, with a greater traffic flow associated with a higher degree of urgency at the junction, and thus a more obvious navigation risk. To estimate the values corresponding to the influence of these three variables on the navigation risk, we used the method of quantitative fuzzy mathematics (see Table 4). Seafarers, shipping corporations, and maritime officers were invited to fill out a questionnaire on the risk ratios of the visibility, route intersection, and channel dimension. The values of these ratios range from 0 to 1. A higher value indicates a greater impact on navigation safety.

**Table 4:** Collection of Quantized Data.

Sample collection site	Visibility	Route intersection	Channel dimension
**Yichang**	0.425	0.525	0.123
**Guizhou**	0.375	0.356	0.123
**Badong**	0.356	0.356	0.215
**Wushan**	0.356	0.285	0.356
**Fengjie**	0.285	0.235	0.356
**Yunyang**	0.356	0.235	0.356
**Wanzhou**	0.425	0.425	0.356
**Zhongxian**	0.634	0.285	0.375
**Fengdu**	0.526	0.356	0.375
**Fuling**	0.735	0.657	0.425
**Changshou**	0.657	0.525	0.735
**Chongqing**	0.853	0.685	0.806
**Jiangjin**	0.853	0.605	0.853

The values of the original variables for each site along the Yangtze River in the Three Gorges Reservoir area from downstream to upstream, as obtained by the survey, statistics, and fuzzy comprehensive evaluation methods, are listed in Table 5.

**Table 5:** Data Acquisition of Control Variables of Navigational Environment.

Site	Values of Control Variables					
	*M*			*N*		
	*x*_*1*_	*x*_*2*_	*x*_*3*_	*x*_*4*_	*x*_*5*_	*x*_*6*_
**Yichang**	0.425	47.0	1.4	0.525	0.123	470.5
**Guizhou**	0.375	43.0	2.4	0.356	0.123	395.2
**Badong**	0.356	44.5	3.0	0.356	0.215	322.5
**Wushan**	0.356	38.0	3.4	0.285	0.356	237.5
**Fengjie**	0.285	45.5	2.8	0.235	0.356	230.6
**Yunyang**	0.356	35.0	3.3	0.235	0.356	228.7
**Wanzhou**	0.425	33.0	3.5	0.425	0.356	248.8
**Zhongxian**	0.634	45.0	3.6	0.285	0.375	236.9
**Fengdu**	0.526	36.0	3.9	0.356	0.375	237.5
**Fuling**	0.735	30.0	4.3	0.657	0.425	246.1
**Changshou**	0.657	34.5	4.5	0.525	0.735	232.6
**Chongqing**	0.853	26.0	5.0	0.685	0.806	249.3
**Jiangjin**	0.853	25.0	5.0	0.605	0.853	179.6

Here, *M* denotes a control variable of the natural environment; *N* denotes a control variable of the traffic environment; *x*_*1*_, *x*_*2*_, and *x*_*3*_ are the original variables of *M*, which respectively represent the visibility, wind, and water velocity; and *x*_*4*_, *x*_*5*_, and *x*_*6*_ are the original variables of *N*, which respectively represent the traffic flow, channel dimension, and route intersection.

### Solution of control variables based on principal component analysis

In the process of obtaining the expressions of *M* and *N*, the original data should be orthogonalized and normalized. In the use of principal component analysis (PCA), the principal components are determined by extracting the first several factors whose variance is greater than one. Further, the variance of the principal component values is the eigenvalue of the corresponding correlation coefficient matrix. The correlation coefficients of each of the two factors are solved on the basis of the standardized data of *x*_1_,*x*_2_,*x*_3_. Then, the correlation coefficient matrix of the three subvariables within the control variable *M* of the natural environment is established (see Table 6).

**Table 6:** Correlation Coefficient Matrix of Natural Environment Control Variable.

Variable	Visibility	Wind	Current
**Visibility**	1.000	-0.735	0.804
**Wind**	-0.735	1.000	-0.859
**Current**	0.804	-0.859	1.000

All the eigenvalues, as well as the contribution rate and accumulative value of the corresponding variance, were acquired using SPSS (19.0). From Table 7, it can be seen that there is only one principal component with a variance greater than one, and the corresponding contribution rate is larger than 85%. This shows that the selected principal component can well represent the information of all the original variables.

**Table 7:** Explained Variance of Natural Environment Control Variable.

Component		1	2	3
**Initial eigenvalue**	**Variance *λ***	2.600	0.272	0.128
	**Variance percentage (%)**	86.670	9.059	4.271
	**Accumulative total (%)**	86.670	95.729	100.000
**Principle component extraction**	**Variance *λ***	2.600		
	**Variance percentage (%)**	86.670		
	**Accumulative total (%)**	86.670		

In order to obtain an expression of the control variables, the correlation coefficient of each variable in the expression should be calculated. First, we need to calculate the correlation coefficient of each variable using the principal component (see Table 8).

**Table 8:** Component Matrix.

Variable	Correlation coefficient (***B***)
**Visibility**	0.907
**Wind**	-0.930
**Current**	0.955

Coefficient *A* of each variable in the principal component is obtained by dividing correlation coefficient *B* by the square root of the eigenvalues of the principal component ($\sqrt{\lambda }$); these *A* values are listed in Table 9.

**Table 9:** Coefficient of Each Variable.

Variable	Coefficient (***A***)
**Visibility**	0.5688
**Wind**	-0.6156
**Current**	0.5456

Thus, the expression of control variable *M* of the natural environment is determined; the expression of control variable *N* of the traffic environment is obtained in the same way (see Eqs 6 and 7, respectively).

$$M=0.5688x_{1}-0.6156x_{2}+0.5456x_{3}$$ (6)

$$N=0.3369x_{4}+0.4736x_{5}-0.4222x_{6}$$ (7)

where *M* denotes the control variable of the natural environment, and *N* denotes the control variable of the traffic environment. *x*_1_,*x*_2_,*x*_3_,…,*x*_6_ are subvariables that are indicators having an influence on the natural environment and traffic environment. They respectively denote the six factors: the visibility, wind force, water velocity, route intersection, channel dimension, and traffic flow.

The calculation and analysis of the variance indicate that *M* and *N* can reflect 86.67% and 96.85%, respectively, of the variable information of the two kinds of environments. The contribution rates for both these control variables are higher than 85%, which indicates a favorable reflection of the information of all the original variables.

From Eqs 6 and 7, we can see that the expression of the principal component *M* comprises three original variables: visibility, wind, and water velocity. These three variables are present in different proportions, among which the absolute value of the coefficient of *x*_*2*_ is the largest, indicating that wind is the dominant factor affecting the natural environment. Similarly, the channel dimension is the dominant factor affecting the traffic environment.

### Simulation of cusp mutation

Upon substituting the normalized values of the variables for the 13 data collection sites in the Three Gorges Reservoir area into Eqs 6 and 7, we obtain comprehensive principal component values of the control variables *M* and *N* in each region, i.e., a comprehensive index (Δ).

**Fig 4:**
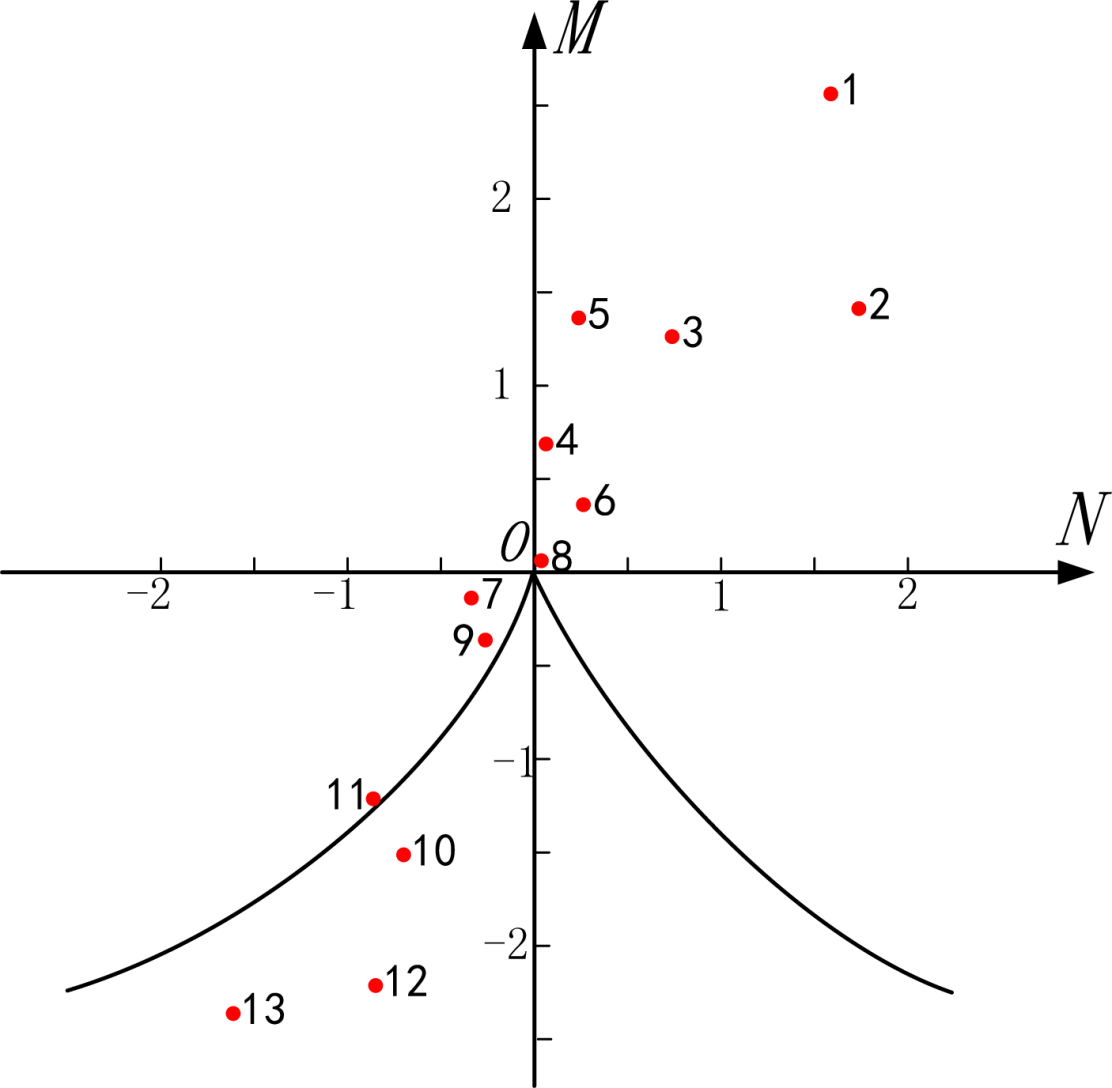
Scattergram of Mutation Situation of Navigational Environment in Three Gorges Reservoir Area. 1, Yichang; 2, Guizhou; 3, Badong; 4, Wushan; 5, Fengjie; 6, Yunyang; 7, Wanzhou; 8, Zhongxian; 9, Fengdu; 10, Fuling; 11, Changshou; 12, Chongqing; 13, Jiangjin.

The values of *M* and *N* for each collection site and the calculation results of Δ are presented in Table 10. On the basis of the values of *M*, *N*, and Δ, the location distribution of each region in the coordinate system of the navigational environment is as shown in Fig 4.

**Table 10:** Score of Navigational Environment Variables and Comprehensive Index of Data Collection Sites along Yangtze River.

Data collection site			Natural environment *M*	Traffic environment *N*	**Δ**
**From downstream to upstream**	**1**	**Yichang**	2.5510	1.5805	200.2464
	**2**	**Guizhou**	1.4145	1.6970	100.3983
	**3**	**Badong**	1.2844	0.7089	30.5171
	**4**	**Wushan**	0.7215	0.0395	3.0469
	**5**	**Fengjie**	1.4323	0.2354	25.0040
	**6**	**Yunyang**	0.3968	0.2181	1.7844
	**7**	**Wanzhou**	-0.1267	-0.2655	1.8869
	**8**	**Zhongxian**	0.0217	0.0052	0.0008
	**9**	**Fengdu**	-0.3695	-0.2298	1.0217
	**10**	**Fuling**	-1.5183	-0.6653	-16.0456
	**11**	**Changshou**	-1.2530	-0.8509	3.8101
	**12**	**Chongqing**	-2.2023	-0.8905	-64.0420
	**13**	**Jiangjin**	-2.3523	-1.5825	-36.5172

It is seen from Table 10 and Fig 4 that the comprehensive evaluation values, i.e., *M* and *N*, of the natural environment and traffic environment gradually decrease from downstream to upstream. Specifically, it is observed that the values of *M* and *N* adjacent to Yichang are the largest, which demonstrates that the natural environment and traffic environment of this region are superior to those of the other regions in the reservoir area owing to a slower water flow and larger channel dimension. In contrast, at Jiangjin, which lies at the end of the reservoir, the comprehensive evaluation values of both the natural environment and traffic environment are lower than those of the other regions because of the influence of the small channel dimension, an increase in obstruction, turbulence of the water flow, and the crossing of routes.

### Zoning of navigation risk assessment

The rules for evaluating the navigational environment according to the characteristics of the cusp catastrophe model are presented in Table 11.

**Table 11:** Evaluation Rules of Navigational Environment.

Δ > 0	Δ = 0	Δ < 0
Safe zone	Transition zone	Danger zone

**Fig 5:**
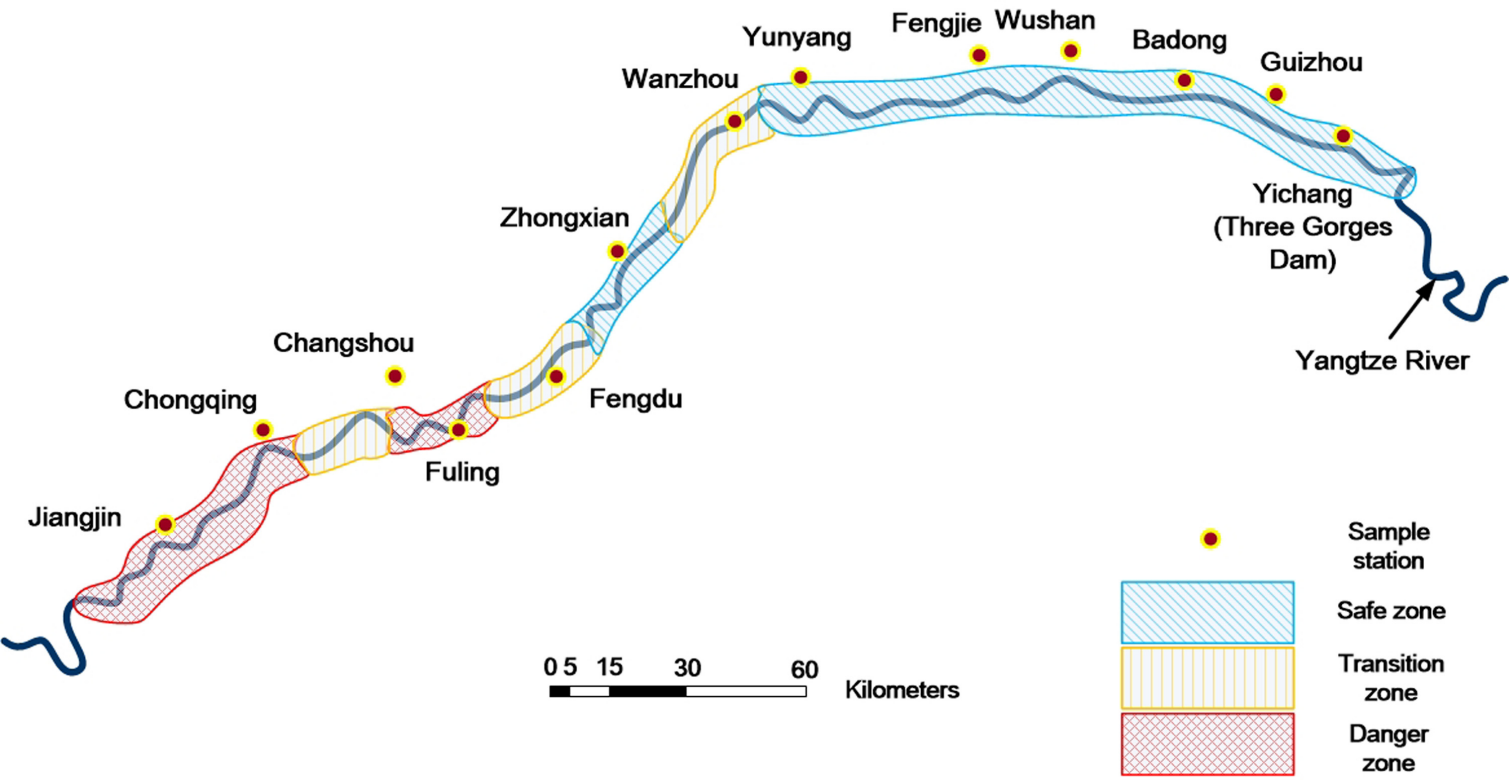
Zoning Map of Navigational Environment in Three Gorges Reservoir Area Prepared from Results of Catastrophe Evaluation. The figure is similar but not identical to the original one, and is shown for illustrative purpose only.

The Three Gorges Reservoir area is zoned on the basis of the results of a catastrophe evaluation; the zoning map is shown in Fig 5.

The following conclusions can be drawn from the results of the mutation risk evaluation, as well as from Fig 5.

- The danger zones (colored red) are scattered mainly in Fuling and the fluctuating backwater region of upper Chongqing. On the one hand, these sections are influenced by fog and wind. On the other hand, during a low-water-level period, the impact of high flow velocities, disordered current, a decrease in the water depth, and an increase in obstructions make the overall situation of the navigational environment unsatisfactory, where the possibility of mutation is the largest. It is worth noting that although Fuling is not in the backwater area, the influence of the water also begins to appear because of the short distance of this area from the backwater area. In addition, this region is one of the three largest hub ports of Chongqing, where the traffic flow and route intersection are more complicated than those in the waters of other regions. The results of the comprehensive evaluation and simulation of the mutation of the navigational environment reveal that the navigational environment of this area is also at risk of mutation, with a higher probability of water traffic accidents.
- The transition zones (colored yellow) are located mainly at Wanzhou, Fengdu, and Changshou. While the traffic flows of these regions are at the same level as those in the downstream segment, the poorer visibility, increased high winds, larger water velocity in the flood period, and narrow shipping lane make the navigational environment in these regions relatively crowded. It is revealed that the situation of the navigational environment is not ideal, but no massive water traffic accidents will occur just yet.
- The safe zones (colored blue) are distributed mostly in the segment from Yichang to Yunyang and Zhongxian. Because of the sufficient water depth and broad waters in this segment, the water velocity is low, and the channel dimension is adequate. The comprehensive evaluation values for both the natural environment and traffic environment at these sites are positive and far from the cusp, indicating that a variation in the navigational environment at these sites will only induce a smooth gradient of the navigation security situation; in other words, the condition of the navigational environment is stable.

According to the accident statistics of the Yangtze River Maritime Bureau (see S1 Table), 58 water traffic accidents occurred in the Three Gorges Reservoir area in the period of 2011 to 2013. Among these 58 accidents, 26 occurred in the backwater area from Changshou to Jiangjin, which is nearly half of the total number of accidents. In the last 3 years, 14 graded water traffic accidents occurred in the reservoir area, of which 8 occurred in the backwater area, which is more than half of the total number. It was shown that the end segment is a high-risk and accident-prone segment. Therefore, it was verified that the fitting rate of the mutation evaluation model is high, and the evaluation results are in sufficient agreement with the actual situation.

### Summary

Water traffic accidents caused by a change in the navigational environment can be summarized as a kind of mutation of the navigation risk caused by the coupling of various factors influencing the navigational environment. In this study, the evolution mechanism of the navigation risk was analyzed by the application of the catastrophe theory. The natural environment and traffic environment were determined to be the control variables by the multivariate statistics method used in combination with principal component analysis. A mutation model of the navigational environment can effectively explain the process of a sudden change in the situation of the navigation safety system caused by the continuous variation of the navigational environment from the viewpoint of the potential for accidents. Δ was used as an evaluation criterion to conduct a risk assessment of the navigational environment at 13 data collection sites along the Yangtze River. The navigation risk was divided into three levels (i.e., zones), according to the evaluation results. The danger zones were scattered mainly in Fuling and the fluctuating backwater region of upper Chongqing; the transition zones were located mainly at Wanzhou, Fengdu, and Changshou; finally, the safe zones were distributed mostly in the segment from Yichang to Yunyang and Zhongxian.

Further, the developed mutation model of the navigational environment was verified to be rational by a comparison of its results with data on the actual accidents that occurred in the Three Gorges Reservoir area from 2011 to 2013. The evaluation results for the navigational environment in each region obtained through the application of the mutation model were essentially in accord with the actual situation. This implied that the developed mutation model can aid in providing valuable data for issuing a warning when the navigation safety in the reservoir area is at risk.

Because the solution of the cusp catastrophe model should be based on a reasonable quantization of the original variables, the values of the original variables directly affect the evaluation results. The navigational environment is a highly complex system that is influenced by various factors. Thus, the topic of how to perform a reasonable and comprehensive quantization of each influencing factor deserves further study.

## Supporting Information

S1 Table Accident Statistics of Three Gorges Reservoir Area in the Period of 2011 to 2013. (DOC)
