# Supplementary material for: Use of Cusp Catastrophe for Risk Analysis of Navigational Environment: A Case Study of Three Gorges Reservoir Area
Source: PLoS One. 2016 Jul 8;11(7):e0158482. doi: 10.1371/journal.pone.0158482 (PMC4938504; doi:10.1371/journal.pone.0158482)
Supplement: S1 Table — (DOC) [file pone.0158482.s001.doc]

**S1 Table. Accident Statistics of Three Gorges Reservoir Area in the Period of 2011 to 2013.**

| **Data collection site** | | | **2011** | **2012** | **2013** | **Total** |
| --- | --- | --- | --- | --- | --- | --- |
| **Downstream↓**  **Upstream** | **1** | **Yichang** | 1 | 0 | 1 | 2 |
|  | **2** | **Guizhou** | 0 | 1 | 0 | 1 |
|  | **3** | **Badong** | 0 | 1 | 1 | 2 |
|  | **4** | **Wushan** | 1 | 0 | 1 | 2 |
|  | **5** | **Fengjie** | 0 | 1 | 0 | 1 |
|  | **6** | **Yunyang** | 1 | 0 | 1 | 2 |
|  | **7** | **Wanzhou** | 2 | 0 | 3 | 5 |
|  | **8** | **Zhongxian** | 1 | 1 | 0 | 2 |
|  | **9** | **Fengdu** | 3 | 0 | 2 | 5 |
|  | **10** | **Fuling** | 5 | 2 | 3 | 10 |
|  | **11** | **Changshou** | 1 | 2 | 0 | 3 |
|  | **12** | **Chongqing** | 6 | 4 | 5 | 15 |
|  | **13** | **Jiangjin** | 4 | 3 | 1 | 8 |
| **Total** | | | 25 | 15 | 18 | 58 |
